# Smart Concrete Using Optical Sensors Based on Bragg Gratings Embedded in a Cementitious Mixture: Cure Monitoring and Beam Test

**DOI:** 10.3390/s24247998

**Published:** 2024-12-14

**Authors:** Edson Souza, Pâmela Pinheiro, Felipe Coutinho, João Dias, Ronaldo Pilar, Maria José Pontes, Arnaldo Leal-Junior

**Affiliations:** 1 Graduate Program in Electrical Engineering, Federal University of Espírito Santo, Vitória 29075-910, ES, Brazil; edson.souza.99@edu.ufes.br (E.S.); maria.pontes@ufes.br (M.J.P.); 2 Bachelor Program in Civil Engineering, Federal University of Espírito Santo, Vitória 29075-910, ES, Brazil; pamela.pinheiro@edu.ufes.br (P.P.); felipe.b.coutinho@ufes.br (F.C.); joao.v.dias@ufes.br (J.D.); ronaldo.pilar@ufes.br (R.P.)

**Keywords:** smart concrete, fiber Bragg grating, shrinkage strain, curing concrete

## Abstract

Smart concrete is a structural element that can combine both sensing and structural capabilities. In addition, smart concrete can monitor the curing of concrete, positively impacting design and construction approaches. In concrete, if the curing process is not well developed, the structural element may develop cracks in this early stage due to shrinkage, decreasing structural mechanical strength. In this paper, a system of measurement using fiber Bragg grating (FBG) sensors for monitoring the curing of concrete was developed to evaluate autogenous shrinkage strain, temperature, and relative humidity (RH) in a single system. Furthermore, K-type thermocouples were used as reference temperature sensors. The results presented maximum autogenous shrinkage strains of 213.64 με, 125.44 με, and 173.33 με for FBG4, FBG5, and FBG6, respectively. Regarding humidity, the measured maximum relative humidity was 98.20 %RH, which was reached before 10 h. In this case, the recorded maximum temperature was 63.65 °C and 61.85 °C by FBG2 and the thermocouple, respectively. Subsequently, the concrete specimen with the FBG strain sensor embedded underwent a bend test simulating beam behavior. The measurement system can transform a simple structure like a beam into a smart concrete structure, in which the FBG sensors’ signal was maintained by the entire applied load cycles and compared with FBG strain sensors superficially positioned. In this test, the maximum strain measurements were 85.65 με, 123.71 με, and 56.38 με on FBG7, FBG8, and FBG3, respectively, with FBG3 also monitoring autogenous shrinkage strain. Therefore, the results confirm that the proposed system of measurement can monitor the cited parameters throughout the entire process of curing concrete.

## 1. Introduction

Smart cities aim to address the challenges presented by the growing trend of migration of people to urban areas through data [1,2]. In this regard, modern society requires a higher demand for civil structures and infrastructures [3,4,5,6]. In this context, structural health monitoring (SHM) plays a key role in the predictive and real-time monitoring of civil structures required in modern urban spaces [7], since such structures are subjected to mechanical loads in conjunction with environmental exposition that can result in damage that deteriorates structural properties over time [8]. For this reason, an important challenge in smart cities is the continuous monitoring of the infrastructure to evaluate the complex mechanical loads generated, crack growth, and corrosion of structural elements [7]. In addition, depending on the severity of the damage, it can be enough to collapse the structure, leading to a loss of a high investment that cannot be recovered and possibly to environmental damage [9].

Concrete is the product of a mixture of water, cement, and aggregate in the correct proportions, produced by hydration reactions between cement and water. At an early stage, particularly during the curing process, concrete develops its potential properties [10,11]. Proper curing is essential for ensuring the quality of the concrete, and if this process is neglected, cracking may occur. However, this is not the only source of early concrete cracking. Autogenous shrinkage is a volumetric contraction that occurs in all cementitious materials due to the chemical equilibrium of the hydration reactions of Portland cement [12]. In other words, the volume of the anhydrous compounds is greater than the volume of the products formed. In general, autogenous shrinkage is small; however, in structures made with a water–cement ratio of less than 0.40, this phenomenon can cause cracking at an early stage, compromising the durability of the structure [13]. Therefore, obtaining information through monitoring from the early stage up to the end of the product’s useful life is essential for developing and maintaining a safe and reliable structure [14]. During the curing process, the main parameters of interest for monitoring are strain, temperature, and humidity [5,6,15]. Among the several types of concrete, reinforced concrete is often used in large buildings and consists of an embedded steel reinforcement; in this case, the best sensor position for the monitoring of a structure manufactured with reinforcing concrete is precisely on the steel reinforcing, in other words, with the sensors embedded in the concrete [16]. Thus, with adequate monitoring of concrete, it is possible to achieve better structural management and a structure with greater mechanical strength and durability [6,14].

Currently, structural health monitoring (SHM) emerges as a promising approach with the boost of technologies such as advanced systems of measurement, in which advances in new sensor technologies play a crucial role. In addition, the application of SHM can generate validation and prediction models to classify the structural state [3,16,17,18]. Nowadays, the main focus of SHM is on small sensor technologies that can be embedded during the manufacturing of different materials and structures. In this way, smart structures can combine both structural and sensing capabilities, potentially increasing the level of sophistication [6,19,20]. In addition, smart structures will generate changes in design and construction approaches, helping to achieve the most efficient and sustainable urban planning and management [21,22].

This demand in the SHM field has attracted the attention of numerous scientists and engineers to optical fiber sensors (OFSs) because of their inherent characteristics. OFSs are light and compact (the fiber diameter is 125 μm) and immune to the corrosion process, which makes these sensors promising for long-term monitoring [23]. These alternative sensors use light modulation as an interrogation method to estimate the parameters of interest. This makes the OFS immune to electromagnetic interference and inherently safe [24]. It is worth mentioning that in an environment that presents considerable electrical noise, e.g., industrial environments, the signal-to-noise ratio (SNR) is significantly affected, interfering with sensor accuracy and consequently making the classification of the structural state more difficult [25,26]. In addition, some OFSs present the capability of multiplexing along the fiber in quasi-distributed or distributed configurations, which increases the measurement field over the structure of interest without the complexity of multiple wires and the necessity of more processing units [27].

Fiber Bragg grating (FBG) sensors are a type of OFS generated by periodic alterations in the core refractive index caused by a UV laser. FBG sensors can be correlated with different physical parameters (e.g. strain, temperature, pressure, and humidity) encoded in the wavelength of reflected light by the gratings. This reflected wavelength is called the Bragg wavelength [28]. FBG sensors present a linear behavior with respect to strain, temperature, and humidity [21,29]. However, for FBG to be sensitive to humidity, a polymer coating (usually polyimide) is added to the region of the sensor element [30,31]. FBG sensors have been applied and evaluated in different areas, from biomedical to industrial and civil infrastructure areas [24,32,33].

Smart concrete may be classified into different types to serve different functions, e.g., self-sensing concrete, self-diagnosis concrete, temperature self-heating concrete, self-curing concrete, bionic self-healing concrete, and ultra-high-performance powder reactive concretes. Thus, in the same way as a smart structure, smart concrete can combine both structural and sensing capabilities. In addition, smart concrete can monitor the curing of concrete, positively impacting design and construction approaches and providing a more secure and reliable structure [34]. Accordingly, FBG sensors were applied to monitor the curing of concrete using a thermocouple and a polymer optical fiber FBG (POF-FBG) humidity sensor, respectively. However, the results of the measured relative humidity by the POF-FBG when the temperature change was significant were not presented [21]. The monitoring of shrinkage strain, creep strain, and elastic strain was performed using FBG sensors [35]. In addition, the real-time monitoring of strain and temperature during the curing of concrete was performed using FBG sensors [15]. In this paper, a system of measurement using FBG sensors for monitoring the concrete from manufacture to final application is proposed. The monitoring of concrete curing was performed to evaluate the autogenous shrinkage strain, temperature, and relative humidity in a single system. Subsequently, a concrete specimen with embedded FBG sensors underwent a bend test simulating beam behavior to evaluate the system of measurement’s capability to make concrete into smart concrete. The monitoring of strain, temperature, and relative humidity was performed from the initial stage of curing up to the seventh day, during which it was possible to measure the relative humidity in the concrete with significant temperature changes occurring. It is worth mentioning that thermocouples were used as a reference for temperature measurements. For comparison purposes, Table 1 presents the available monitored data for parameters of interest (strain, temperature, and relative humidity) related to the proposed system and the systems of other works cited here.

As shown in Table 1, the proposed system is the only one that presents the advantage of multiparametric analysis, in this case, autogenous shrinkage strain, temperature, and relative humidity in a single system. It is very interesting because the monitored parameters may be correlated to evaluate the impact of different types of cures on the mechanical properties of smart concrete. In addition, the sensitivity of the systems of measurements related to monitored parameters is also highlighted.

## 2. Materials and Methods

### 2.1. Fiber Bragg Gratings

The periodic modulations in the core refractive index presented by the FBG sensors reflect a small portion of the signal injected in the core of the optical fiber. This small portion of the signal is centered at a specific wavelength known as the Bragg wavelength, $\lambda_{Bragg}$ [28]. The Bragg wavelength is reflected in Equation (1).

(1) $$\lambda_{Bragg}=2n_{eff}\Lambda$$

$n_{eff}$ is the core effective refractive index modulated by the UV lasers during the inscription process, and Λ is the spatial periodicity of the gratings generated. In this way, the measurand of interest is encoded in the Bragg wavelength.

**Figure 1:**
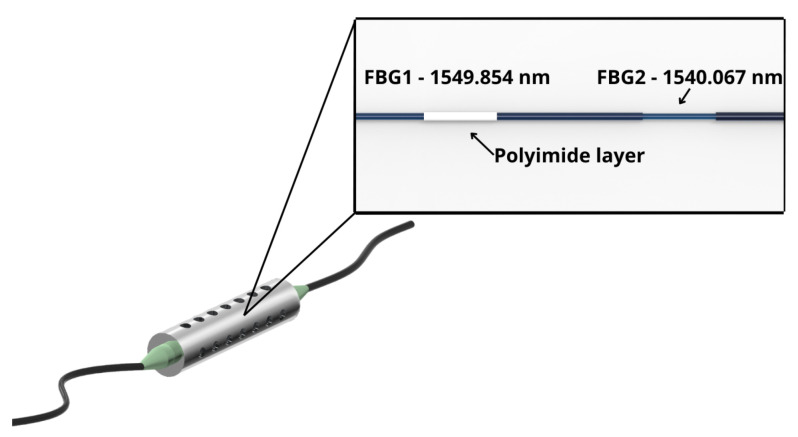
FBG Hygrometer Sensor. The zoomed-in view shows details of the FBGs.

**Figure 2:**
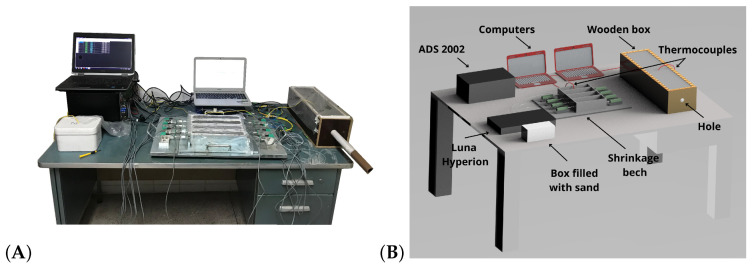
The curing concrete setup photo is presented in (**A**). The experimental components are identified in the schematic drawing (**B**).

In this work, FBG1 and FBG2, which are related to the FBG Hygrometer Sensor, were used to monitor the relative humidity and temperature, respectively. FBG3 up to FBG6 were used to monitor the autogenous shrinkage strain. The FBG4, FBG7, and FBG8 were used in the beam test. It is worth mentioning that all tests were performed without FBG sensors’ loss of signal. In addition, the original Bragg wavelengths of FBG1-2 and FBG3-6 are shown in Figure 1 and Figure 2, respectively.

#### 2.1.1. FBG Strain Sensor

The measurand of interest encoded in the Bragg wavelength can be estimated by observing the behavior of the sensor, in this case, the FBG sensors’ sensitivity to the physical parameters (strain, temperature, and relative humidity). In this work, the adopted sensitivity values to the strain and temperature of FBG were 1.20 pm/με and 9.40 pm/°C, respectively, according to commercial FBGs, which are values widely found in the literature [36]. However, it is worth mentioning that the experimental strain and temperature calibration of FBG sensors provide results with higher accuracy. FBG sensors suffer from cross-sensitivity, in which two or more parameters influence the Bragg wavelength shift. To the FBG strain sensor, the cross-sensitivity to strain and temperature may be mathematically interpreted by Equation (2) [37].

(2) $$\frac{\Delta \lambda_{Bragg}}{\lambda_{Bragg}}=\left(1-P_{e}\right)\Delta \varepsilon +\left(\alpha +\zeta \right)\Delta T$$

In Equation (2), $P_{e}$ is the photoelastic coefficient, α is the thermal expansion coefficient, ζ is the thermal optic coefficient, and $\Delta \lambda_{Bragg}$, Δε, and ΔT are the Bragg wavelength and strain and temperature shift experimented by the sensor, respectively [37].

#### 2.1.2. FBG Hygrometer Sensor

An FBG Hygrometer Sensor was used to measure temperature and relative humidity. This sensor is designed with two FBGs multiplexed in a single optical fiber, in which one FBG is covered by a polyimide layer to measure relative humidity. The polyimide presents the capability of absorbing and desorbing moisture quickly and has good linear expansion characteristics. This polyimide cover is preferred in complex applications, and in this case, the present work presents variations in the relative humidity and temperature of the sensor simultaneously [38]. This optical fiber is encapsulated with a steel cylinder, as shown in Figure 1.

According to Figure 1, the Bragg wavelength of relative humidity FBG (FBG1) is 1549.854 nm, while the Bragg wavelength of temperature FBG (FBG2) is 1540.067 nm. This sensor presents the cross-sensitive effect caused by the temperature and relative humidity at FBG1, which may be represented by Equation (3) [30].

(3) $$\Delta \lambda_{Bragg}=S_{RH}\Delta RH+S_{T}\Delta T$$

$S_{RH}$ and $S_{T}$ are relative humidity and temperature sensitivity, and $\Delta \lambda_{Bragg}$, ΔRH, and ΔT are the shifts in the Bragg wavelength, relative humidity, and temperature, respectively. FBG1 presents the linear behavior caused by the polyimide layer absorbing water from the environment, causing mechanical strain in the Bragg gratings [30]. The temperature was compensated using FBG2. In addition, a calibration test was performed to obtain the temperature and relative humidity sensitivity to FBG1 and the temperature sensitivity to FBG2.

### 2.2. Calibration Test

The calibration test consisted of positioning the FBG Hygrometer Sensor in a climatic chamber (Quimis, BR) with temperature and relative humidity control. The relative humidity inside the chamber was varied to 60 %RH, 70 %RH, 80 %RH, 90 %RH, and 95 %RH, while the temperature was kept constant. This process of varying the relative humidity was performed at three different temperature values, 30 °C, 35 °C, and 45 °C. It is worth mentioning that the Bragg wavelength was acquired by the commercial interrogator of the sensor only for 2 min in each step. In this way, it was possible to obtain three values for the relative humidity sensitivity for FBG1 and five values for the temperature sensitivity for FBG1 and FBG2 related to the FBG Hygrometer Sensor.

### 2.3. Concrete Mixture

In this study, the use of self-compacting concrete was employed as a means of preventing fiber rupture during the compaction process. In addition, the coarse aggregate was not utilized due to the mold dimensions. A high-initial-strength Portland cement, natural quartz sand, and a polycarboxylate-based superplasticizer were used, with the mix design based on the study by De Matos et al. [39]. To focus on the evaluation of autogenous shrinkage, the water/cement ratio was reduced to 0.30. The superplasticizer content was adjusted to achieve a mini-flow spread diameter of 240–260 mm, as recommended by EFNARC [40] for self-compacting mixes. This resulted in a mass proportion of cement:sand:water:superplasticizer of 1:1.5:0.30:0.33%. Following the mini-flow test, two types of prismatic specimens were cast: one 150 × 150 × 450 mm specimen to monitor temperature and relative humidity, and four 50 × 50 × 285 mm specimens to evaluate autogenous shrinkage strain.

### 2.4. Monitoring Curing Concrete

#### Experimental Setup

The experimental setup presented in Figure 2 was developed to monitor the autogenous shrinkage strain, temperature, and relative humidity from the early stage up to the 7th day.

The black box presented in Figure 2 represents the ADS 2002 AD Converter and the Luna Hyperion interrogator, Luna, Chamblee, Georgia, USA which were both set to a sampling frequency of 1 Hz. A reference K-type thermocouple was positioned in the white box filled with sand to thermally isolate this sensor that was utilized to monitor the temperature room. Two other K-type thermocouples were used to measure the temperature in the concrete specimen cast in the wooden box and on the shrinkage bench, respectively. In addition, two computers were necessary to develop the system of measurement to communicate with the ADS 2002 and the Luna Hyperion interrogator.

The first concrete specimen was cast in a wooden box, presented in Figure 2, which was fabricated with a hole where the FBG Hygrometer Sensor was embedded to share the same environment as this specimen. In this way, the FBG Hygrometer Sensor would not suffer any damage, and the effects of temperature and relative humidity would be effectively isolated due to the absence of mechanical strain.

**Figure 3:**
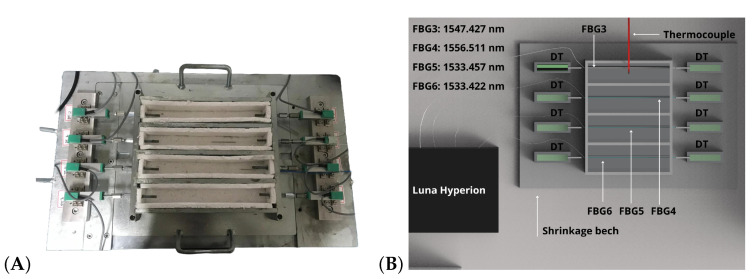
The photo (**A**) presents the shrinkage bench, where the autogenous shrinkage strain was measured. The distribution of the FBG strain sensors is identified in the schematic drawing (**B**).

The other four concrete specimens were launched on the shrinkage bench, where four FBG strain sensors were positioned as shown in Figure 3, before the concrete was poured.

It is worth mentioning that the FBG strain sensors were pre-tensioned for positioning, and a polytetrafluoroethylene (PTFE) cover was added on FBG3, while the other optical fibers were bare. The PTFE cover was added to provide greater mechanical strength for the optical fiber because the FBG strain sensors are physically in contact with the concrete. In addition, the choice of material was also based on strain transfer to the optical fiber, ensuring that the effects of autogenous shrinkage strain could be evaluated through the sensors’ signal. For this reason, materials with greater stiffness, e.g., steel and ceramic, were not considered because strain transfer may not be effective in evaluating the effects of autogenous shrinkage. The inner wall of the shrinkage bench was covered by polystyrene to prevent constraint in the concrete. In addition, displacement transducers (DTs) may be integrated as strain references in the system of measurement proposed, as shown in Figure 3.

### 2.5. Beam Test

A universal test machine (Instron, EUA) was used to excite the concrete specimen through the application of load cycles. This universal test machine has a control system for load, strain, and displacement. The load cycles were applied in a force range of 100–900 N.

#### Experimental Setup

**Figure 4:**
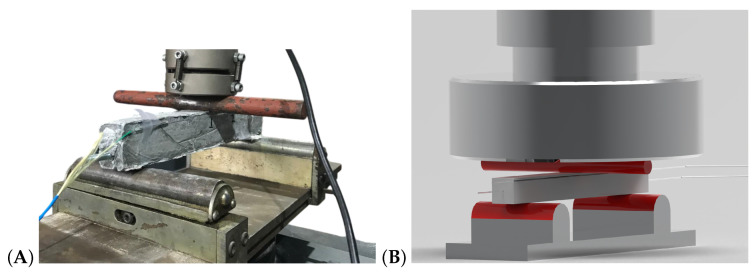
The bend test using three-point bending. (**A**) presents a photo of the positioning of smart concrete and (**B**) presents a schematic representation using two supports and one loading point, highlighted in red.

To evaluate the capability of FBG sensor to transform the concrete element in a smart structure, one concrete specimen of shrinkage bench (specimen with FBG4) underwent a bend test after of 7 days of curing. This bend test was performed using two supports and one loading point (three-point bending), as shown in Figure 4.

The red parts in Figure 4B indicate the supports and the load points, where force is induced by the movement of the loading frame. To obtain a more robust evaluation, two additional FBG strain sensors were bonded to the surface of the beam. No temperature compensation on the readings was needed as tests were conducted in a temperature-controlled room. In addition, data sampling was performed at 50 Hz for this test due to the imposed cyclic loading.

## 3. Results and Discussions

### 3.1. Calibratrion Test

**Figure 5:**
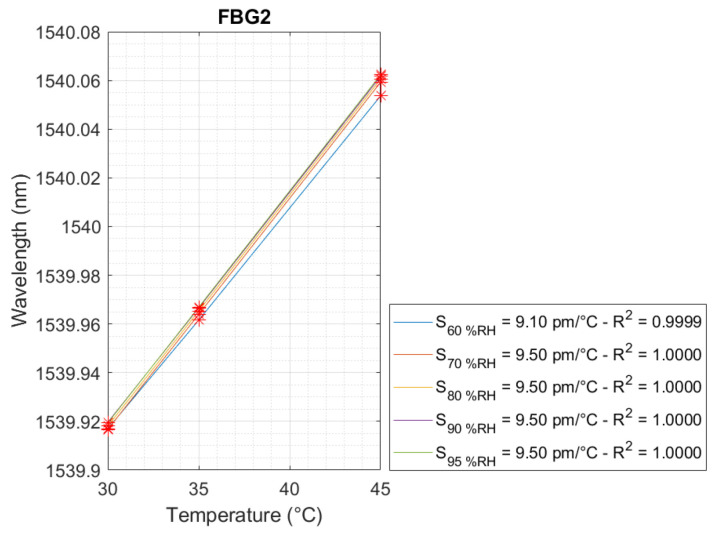
Temperature sensitivity calibration for FBG2 for relative humidities of 60 %RH, 70 %RH, 80 %RH, 90 %RH, and 95 %RH.

**Figure 6:**
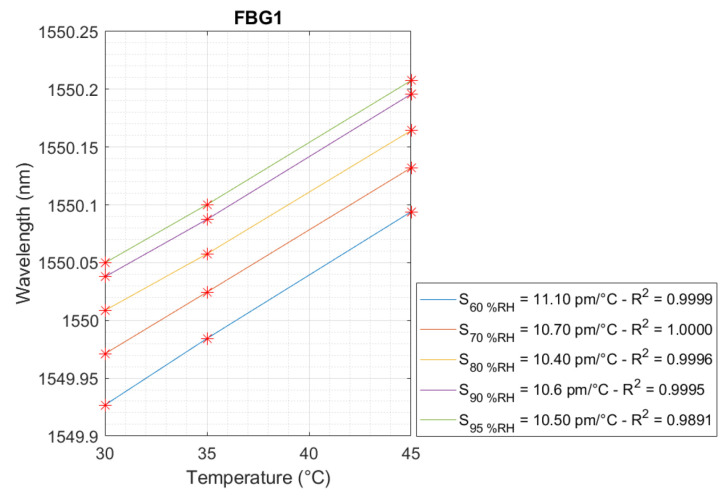
Temperature sensitivity calibration for FBG1 for relative humidities of 60 %RH, 70 %RH, 80 %RH, 90 %RH, and 95 %RH.

**Figure 7:**
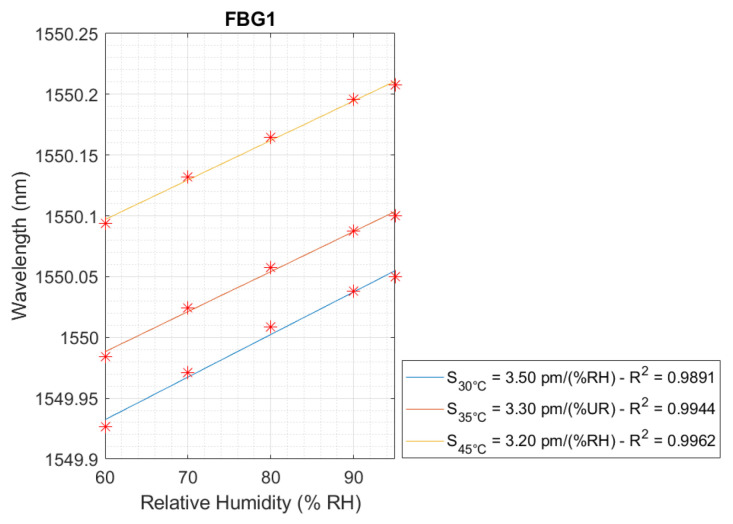
Relative humidity sensitivity calibration for FBG1 for temperatures of 30 °C, 40 °C, and 45 °C.

The linear behavior of temperature and relative humidity is presented in Figure 5, Figure 6 and Figure 7, respectively, for FBG2 and FBG1. Regarding the temperature, three linear curves were achieved for FBG1 and FBG2, and five linear curves for FBG1 with respect to the relative humidity for the FBG Hygrometer Sensor. Thus, the temperature and relative humidity sensitivity are presented in each curve.

In Figure 5, the linear curves of FBG2 presented a high adjustment coefficient, R², in which the minimum and maximum values for this parameter were 0.9999 and 1.0, respectively. FBG2 presented a temperature sensitivity of 9.50 pm/°C for relative humidities of 70 %RH, 80 %RH, 90 %RH, and 95 %RH, while for a relative humidity of 60 %RH, the temperature sensitivity was 9.10 pm/°C. In this case, the temperature sensitivity of 9.50 pm/°C was selected to represent the temperature sensitivity of FBG2.

As shown in Figure 6, the linear curves of FBG1 presented variation in temperature sensitivity in a range of 10.50–11.10 pm/°C. In this case, the minimum and maximum values of R² were 0.9891 and 1.0, respectively. The temperature sensitivity of 10.66 pm/°C was selected based on the average temperature sensitivity value to represent the temperature sensitivity for FBG1.

Accordingly, Figure 7 presents the linear curves regarding relative humidity sensitivity of FBG1 whose values were of 3.50 pm/%RH, 3.30 pm/%RH, and 3.20 pm/%RH, at 30 °C, 35 °C, and 45 °C, respectively. Thus, to represent the relative humidity sensitivity in the concrete curing monitoring test, the average value of 3.33 pm/%RH was selected. In addition, the highest R² value was 0.9962.

### 3.2. Concrete Curing Monitoring Test

The multiparametric simultaneous monitoring of the curing concrete can be evaluated in detail by three sources of information, in this case, autogenous shrinkage strain, temperature, and relative humidity. Thus, the curing process can be optimized based on these parameters to obtain smart concrete with the potential properties. However, due to the weakness of the optical fiber and the physical contact necessary with the concrete, the FBG strain sensor of the developed system does not present durability under harsh conditions related to higher strain. But this can be easily mitigated by encapsulating the FBG strain sensor in the system. On the other hand, there is no contact between the FBG Hygrometer Sensor and the concrete, which means the integrity of the sensor is maintained, and in the final of curing process, this sensor is immediately ready to be used again. The following results related to temperature and relative humidity pertain to the concrete specimen inside the wooden box presented in Figure 2. The results related to autogenous shrinkage strain are relative to the concrete specimen on the shrinkage bench presented in Figure 3. It is worth mentioning that the initial temperature and relative humidity were 24.2 °C and 57.4 %RH, respectively.

#### 3.2.1. Temperature and Relative Humidity

**Figure 8:**
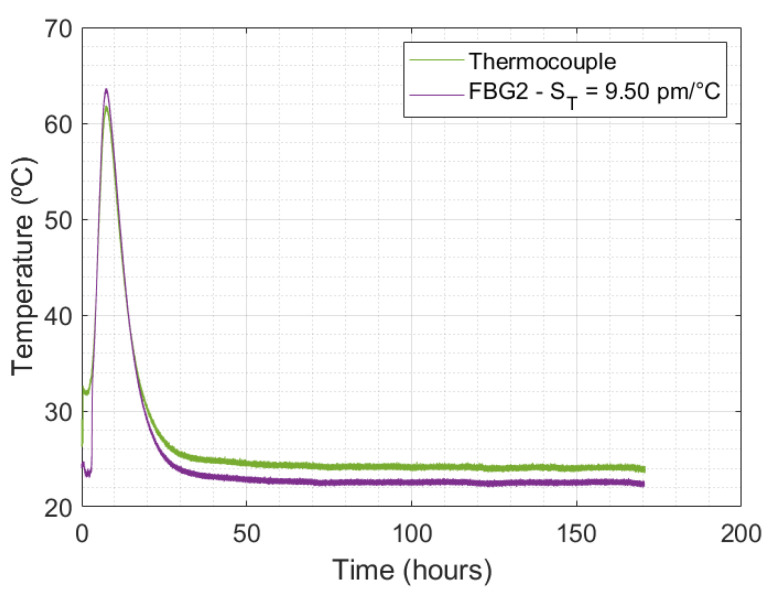
Comparison between the temperature curves obtained by the thermocouple and FBG2.

In the concrete specimen inside wooden box, the monitoring temperature made by FBG2 related to the FBG Hygrometer Sensor and a thermocouple is presented in Figure 8, which shows the temperature evolution over time.

As shown in Figure 8, at the 10 h mark, an expected maximum temperature was measured by both sensors. The maximum values for the thermocouple and FBG2 were 63.65 °C and 61.85 °C, respectively. The hydration reaction from the cement was exothermic, which led to this increase in temperature. This heating was observed for up to 50 h, after which the temperature measurements become nearly constant. In addition, the maximum relative error between the sensors was 9.27%.

**Figure 9:**
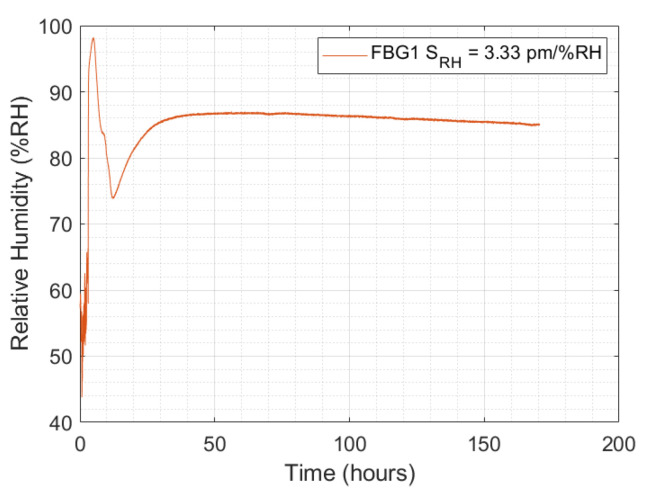
Relative humidity variation versus the test time, monitored by FBG1.

The monitored relative humidity by the FBG Hygrometer Sensor is presented as a relative humidity evolution over time, as shown in Figure 9.

This curve presents a maximum value of relative humidity before 10 h of 98.20 %RH, after which the relative humidity decreases to about 74 %RH and increases again to decrease at a lower rate. This behavior, with a quick decrease in the relative humidity, can be related to some defect in the sealing of the hole made to position the FBG Hygrometer Sensor. In this work, the hole was sealed on both sides with polystyrene covered with aluminum foil.

#### 3.2.2. Autogenous Shrinkage Strain

**Figure 10:**
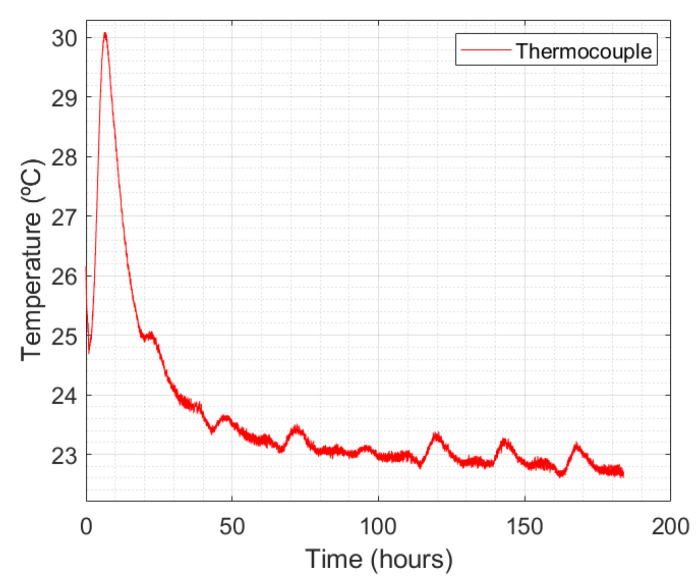
Monitored temperature by thermocouple versus time in the concrete specimen of shrinkage bench.

The FBG strain sensors embedded in the concrete suffer from the effect of cross-sensitivity caused by the strain and temperature, as shown in Equation (3), due to simultaneous changes in these parameters. The temperature curve over time is presented in Figure 10 and was estimated by a thermocouple to perform the temperature compensation in the FBG strain sensor.

In this temperature curve, the same behavior can be observed related to the temperature in the concrete specimen in the wooden box. At about 10 h, there was an increase in temperature and after about 50 h, the temperature measured values were practically constant. However, the maximum temperature value was 30.44 °C. The difference between the maximum measured temperature values is related to the volume of different specimens. As the volume of specimens on the shrinkage bench is lower, the thermal inertia is also lower.

**Figure 11:**
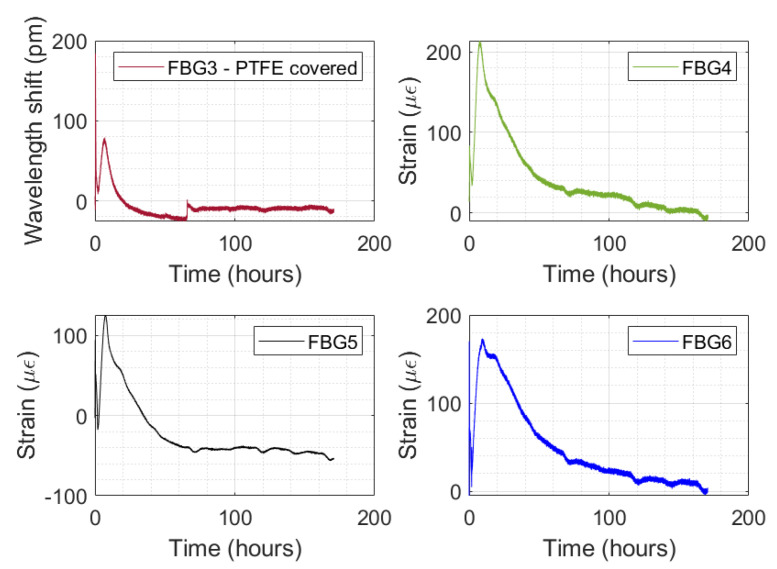
Monitored strain by the FBG4, FBG5, and FBG6 strain sensors versus time. For FBG3, the Bragg wavelength shift during the test is presented.

The strain monitored by the FBG strain sensors is presented in Figure 11, with the time on the abscissa axis. It is worth mentioning that the adopted strain sensitivity to the FBG strain sensor (FBG4, FBG5, and FBG6) was 1.20 pm/με. For the FBG3 with the PTFE cover, only the wavelength shift versus time is presented. To estimate the strain by FBG3, a process of strain characterization to obtain the strain sensitivity is necessary. However, the signal of FBG3 is also evaluated.

The strain curve in Figure 11 (FBG4, FBG5, and FBG6) does not represent the autogenous shrinkage strain yet. The increase in strain observed in the curve indicates a traction strain in the optical fiber related to the concrete laying on the shrinkage bench. It is interesting because this concrete laying time can be estimated through the proposed system of measurement. The maximum values of monitored strain by FBG4, FBG5, and FBG6 were 213.64 με, 125.44 με, and 173.33 με. In addition, the PTFE-covered FBG presented a consistent signal, showing behavior similar to the other FBG strain sensors. This suggest that a PTFE cover may be added to the optical fiber to increase the mechanical strength of FBG strain sensors.

**Figure 12:**
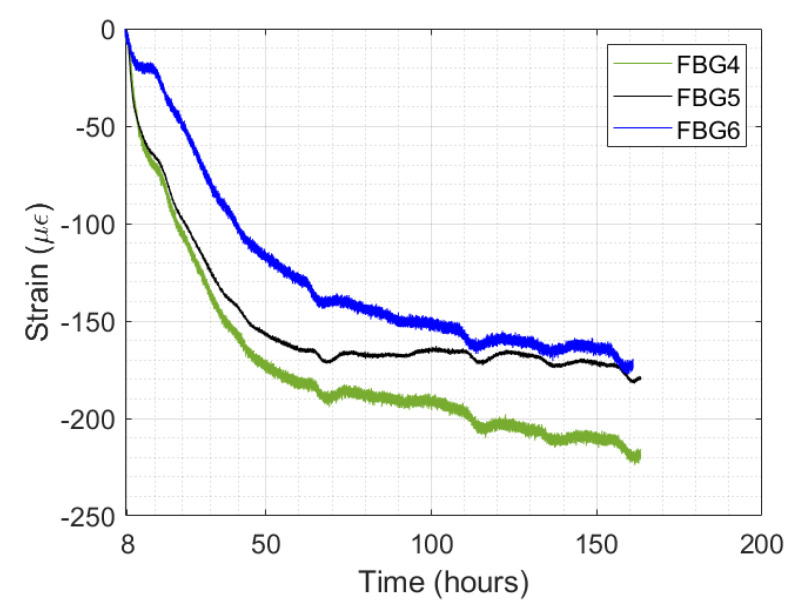
Monitored autogenous shrinkage strain by FBG4, FBG5, and FBG6 versus test time.

The autogenous shrinkage strain is the monitored strain after the maximum point (at 8 h), as presented in Figure 11. The autogenous shrinkage strain curve over time is presented in more detail in Figure 12.

The FBG strain sensors present a similar behavior strain curve to each other, with the strain curve approaching a horizontal asymptote. This horizontal asymptote is more pronounced in FBG5. In this case, the FBG strain sensors act as a point sensor; then, the different measured values of autogenous shrinkage strain were likely due to the distribution of the sensors on the shrinkage bench. In addition, the maximum autogenous shrinkage strain recorded by FBG4, FBG5, and FBG6 were 223.89 με, 182.12 με, and 177.51 με, respectively.

### 3.3. Beam Test

**Figure 13:**
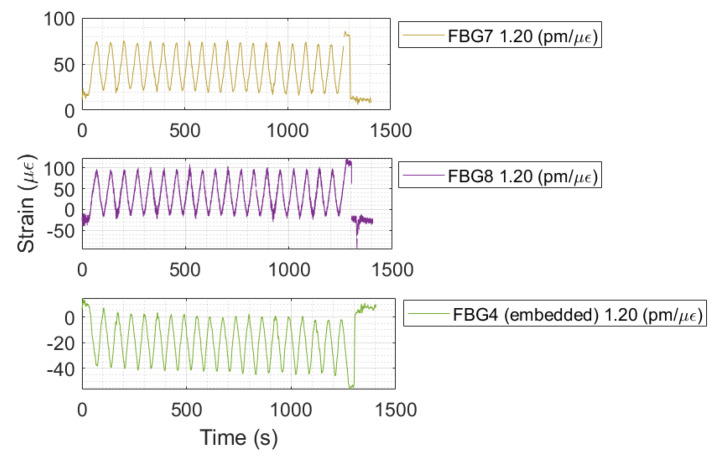
FBG7, FBG8, and FBG3 strain responses to applied bend load cycles.

To evaluate the response of the FBG strain sensor that was embedded with the capability to transform the concrete specimen in a smart structure, the strain curve of FBGs positioned on the surface are shown together with the strain curve of embedded FBGs, as presented in Figure 13.

As the beam test consisted of applying bend load cycles, the FBGs sensors presented senoidal behavior. The response of the embedded FBG strain sensor was 180° out of phase with FBG7 and FBG8, as may be observed. This is explained by the fact that FBG7 and FBG8 were positioned on the other side of the neutral axis present during beam bending. Thus, while FBG4 experienced traction, the other two FBGs experienced compression. The maximum strain measurements were 85.65 με, 123.71 με, and 56.38 με for FBG7, FBG8, and FBG4, respectively, in which FBG4 also monitored the autogenous shrinkage strain. The beam test was conducted without any signal loss from the embedded FBGs during the load cycles. This demonstrates that the FBG sensor is a real and potential alternative for transforming regular concrete into a smart concrete structure through the system of measurement developed.

## 4. Conclusions

This work presented the application of FBG sensors from the manufacturing stage up to the final application of concrete, transforming regular concrete into a smart concrete structure. The system of measurement developed was able to monitor the curing concrete with significant variations in temperature, in which the measured maximum values by the FBG Hygrometer Sensor were 63.65 °C and 98.20 %RH. Autogenous shrinkage strain was also monitored by the system. The measured maximum autogenous shrinkage strain values were 223.89 με, 182.12 με, and 177.51 με, for FBG4, FBG5, and FBG6, respectively. The beam test was conducted without any loss of signal of embedded FBG during the load cycles, with the maximum strain measurements of 85.65 με, 123.71 με, and 56.38 με for FBG7, FBG8, and FBG4, respectively. It is worth mentioning that FBG4 also monitored autogenous shrinkage strain. In addition, the integrity of the sensors was maintained throughout all tests. These results show the FBG sensor is a real and potential alternative for transforming concrete into a smart structure through the system of measurement developed, monitoring the entire curing process of concrete. The system of measurement is also able to obtain the concrete laying time by detecting traction strain in the optical fiber. For future work, the multiparametric analysis provided by this system of measurement can be used to correlate the monitored parameters in different types of cure with the mechanical properties obtained by the smart concrete in the final step of this process. In addition, the system can be optimized for applications in the field. However, as optical fibers present considerable weakness, a prior evaluation of the involved strains is necessary to develop proper encapsulation and maintain the signal’s integrity during operation.

## Figures and Tables

**Table 1:** Monitored parameters through the system developed in this work and in related works. Souza et al.: strain, temperature, and relative humidity; Theoudosiou et al. [21]: relative humidity; Yaszdizadeh et al. [35]: strain; Jo et al. [15]: strain and temperature.

	Strain	Temperature	Relative Humidity
Souza et al.	1.20 pm/με	9.50 pm/°C	3.33 pm/%RH
Theodosiou et al. [21]			18.87 pm/%RH
Yaszdizadeh et al. [35]	1.20 pm/με		
Jo et al. [15]	1.10 pm/με	28.50 pm/°C	

## Data Availability

The raw data supporting the conclusions of this article will be made available by the authors on request.
